# Characterization of Adeno-Associated Virus Capsid Proteins with Two Types of VP3-Related Components by Capillary Gel Electrophoresis and Mass Spectrometry

**DOI:** 10.1089/hum.2021.009

**Published:** 2021-11-15

**Authors:** Hiroaki Oyama, Kentaro Ishii, Takahiro Maruno, Tetsuo Torisu, Susumu Uchiyama

**Affiliations:** ^1^ Department of Biotechnology, Graduate School of Engineering, Osaka University, Suita, Japan.; ^2^ Exploratory Research Center on Life and Living Systems (ExCELLS), National Institutes of Natural Sciences, Myodaiji-cho, Japan.

**Keywords:** adeno-associated virus, viral protein, capillary gel electrophoresis, mass spectrometry

## Abstract

Recombinant adeno-associated virus is a leading platform in human gene therapy. The adeno-associated virus (AAV) capsid is composed of three viral proteins (VPs): VP1, VP2, and VP3. To ensure the safety of AAV-based gene therapy products, the stoichiometry of VPs of AAV vector should be carefully monitored. In this study, sodium dodecyl sulfate–polyacrylamide gel electrophoresis, capillary gel electrophoresis (CGE), and liquid chromatography–UV–mass spectrometry (LC-UV-MS) were performed to evaluate the VP components of AAV1, AAV2, and AAV6. Two types of VP3-related components, VP3 variant and VP3 fragment, were identified. The VP3 variant was the N-terminal shorter VP3, of which the translation started at M211, not at the conventional initiation codon, M203. The VP3 variant could be generated by leaky scanning of the first initiation codon of VP3. We also showed that the VP3 variant was identified in a minor peak before VP3 in CGE measurement. Meanwhile, the VP3 fragment was the C-terminal cleaved VP3, of which the sequence of VP3 ended at D590 or D626, indicating that cleavage occurred between D590 and P591, or D626 and G627. The cause of the cleavage of the DP or DG sequence was hydrolysis due to low pH of the mobile phase and high temperature of the column oven in the LC system, which was necessary to clearly separate the peak of VPs. VP3 fragments, detected only in LC-UV-MS in small amount account with less than 3% of total peak area, should be included in the quantification of VP3. Finally, the relationship of VP stoichiometry determined by the above three methods was discussed. From this study, we proposed that the VP components of AAV should be complementarily evaluated by CGE and LC-UV-MS.

## Introduction

Adeno-associated virus (AAV) is a nonenveloped virus belonging to the genus *Dependoparvovirus* in the family *Parvoviridae*.^1–3^ The AAV particle is composed of an icosahedral AAV capsid and a single-strand DNA of ∼4.7 kb. The genome contains two open reading frames for the Rep gene and Cap gene between inverted terminal repeat sequences. The Rep gene encodes the nonstructural proteins Rep78, Rep68, Rep52, and Rep40, which are responsible for viral genome replication and packaging. The CAP gene produces viral proteins (VPs): VP1, VP2, and VP3, which assemble to form the *T* = 1 icosahedral capsid consisting of 60 VPs. The VP composition of the AAV capsid is estimated to be a molar ratio of 1:1:10,^4,5^ indicating that one AAV capsid likely has 5 VP1, 5 VP2, and 50 VP3. In the open reading frame of the CAP gene, there is an alternative open reading to express an assembly-activating protein, which is essential for capsid assembly.^6^

As the basic biology of AAV has been investigated, recombinant AAV (rAAV) vector has been utilized as a gene delivery vector in human gene therapy.^2,3,7^ AAV has multiple serotypes showing different tissue tropism,^8,9^ therefore, it is possible to design the rAAV vector to deliver the target gene to a specific tissue. Several therapeutic rAAV products have already been approved for human gene therapy: Glybera (uniQure) is an AAV1 product for lipoprotein lipase deficiency approved by the European Medicines Agency (EMA) in 2012 (the marketing was withdrawn in 2017), Luxturna (Spark Therapeutics) is an AAV2 product for retinal dystrophy approved by the U.S. Food and Drug Administration (FDA) in 2017 and subsequently by the EMA in 2018, and Zolgensma (Novartis) is an AAV9 product for spinal muscular atrophy approved by the FDA in 2019. In addition, over 100 clinical trials involving AAV-based gene therapy products are ongoing.^2^

To ensure the safety and efficacy of rAAV drugs for human gene therapy, the quality of AAV vector should be carefully monitored and controlled.^10,11^ Bosma *et al.* suggested that the VP ratio of the capsid influences the vector potency of AAV5 produced by the baculovirus system.^12^ Considering that the unique sequence of VP1 has a phospholipase A2 domain essential for endosomal escape,^13,14^ the lower amount of VP1 could decrease the transduction efficiency. Also, process or product-related degradations of VPs could decrease vector potency and have a risk of immunogenicity.^15,16^ Therefore, the VP components of the AAV capsid should be carefully monitored for quality control and various methods have been attempted to characterize.

Until now, VP stoichiometry has been conventionally evaluated by electrophoresis method, especially sodium dodecyl sulfate–polyacrylamide gel electrophoresis (SDS-PAGE) in which silver staining or fluorescent staining using SYPRO^®^ Ruby dye improved the sensitivity for detection of VP bands.^17,18^ Although staining with dyes and relationship between band intensity and actual protein amounts are required for elucidating the stoichiometry, the relationship for VPs has yet to be determined. Capillary gel electrophoresis (CGE), which has now become a major method for the quality control of biopharmaceuticals, also suggested excellent sensitivity and separation on the VPs.^19,20^ Separated proteins are detected by absorption of peptide bonds or aromatic amino acid residues without staining, therefore, direct quantifications of VPs could be achieved. In addition, as introduced later, electrophoresis-based methods detected the bands/peaks corresponding to components other than VP1, VP2, and VP3, while these bands/peaks were unidentified.

Mass spectrometry-based methods have been widely used for characterizing biopharmaceuticals.^21–23^ Also in AAV, mass spectrometry methods have been recently applied for structural analysis of VPs. Intact mass spectrometry (intact MS) measurement combined with peptide mapping results revealed that the N-terminal methionine residue was cleaved and the next alanine residue was acetylated in VP1 and VP3, whereas there was no acetylation in VP2 for multiple AAV serotypes (AAV1, AAV2, AAV5, AAV7, AAV9, and AAVrh10).^24^ Another study employing a hydrophilic interaction chromatography for separation of VPs for intact MS showed that the VP ratio and the post-translational modification (PTM) level varied among AAV8 samples produced in different lots.^25^ Intact MS combined with LC separation can provide confirmation of therapeutic mass of VPs, however, there are still a few studies that focus on quantification of VPs or characterization of VP isoform.

As the characterization methods of VPs have been developed, several researches suggested the presence of VP-related components, which were different from conventional VPs.^20,24–27^ However, these components were neither fully identified nor quantified. Appropriate quantification of VP-related components as well as VP1, VP2, and VP3 is necessary for better quality control of AAV-based gene therapy products. In the present study, SDS-PAGE, CGE, peptide mapping analysis, and liquid chromatography–UV–mass spectrometry (LC-UV-MS) were used to characterize all VP components derived from AAV vector, where the sequences identified from LC-UV-MS were employed for the analysis of CGE results. We identified two nonconventional components of VP3, VP3 variant and VP3 fragment.

In this study, the VP3 variant was detected in both peptide level and protein level, providing strong evidence of existence. In addition, VP3 variant was identified in a minor peak before VP3 in CGE measurement. Meanwhile, the cleavage sites of VP3 that produced VP3 fragments were identified. These VP3 fragments were only observed in LC-UV-MS. Finally, the ratios of VPs, including the VP3 variants and VP3 fragments determined by SDS-PAGE, CGE, and LC-UV-MS, were compared and the relationship among the three methods was discussed. We concluded that VP components of AAV should be evaluated complementarily by CGE and LC-UV-MS measurements.

## Experimental

### Materials

AAV1 (5.82 × 10^12^ vg/mL), AAV2 (1.46 × 10^12^ vg/mL), and AAV6 (3.85 × 10^12^ vg/mL) samples were provided by the Manufacturing Technology Association of Biologics (Tokyo, Japan). All AAV samples were formulated in 1× PBS at pH 7.4 (Thermo Fisher Scientific, Waltham, MA, USA).

### Methods

#### Sodium dodecyl sulfate–polyacrylamide gel electrophoresis

AAV samples were diluted with a solution mixed 4 × lithium dodecyl sulfate sample buffer (Thermo Fisher Scientific) and 10 × reducing agent (Thermo Fisher Scientific) at 5:2 ratio. The mixtures containing the AAV samples (∼2.0 × 10^10^ vg) were incubated for 10 min at 65, 75, and 80°C for AAV2, AAV6, and AAV1, respectively. The heating temperature was determined from the conformational stability of the capsid. The samples and ladder solution (BenchMark™ Protein Ladder; Thermo Fisher Scientific) were loaded to a 4–12% Bis Tris gel (Thermo Fisher Scientific) and run in 1 × 3-(N-morpholino)propanesulfonic acid buffer (Thermo Fisher Scientific) for 100 min at a constant voltage of 100 V. SYPRO^®^ Ruby dye (Thermo Fisher Scientific) was used for staining the gel. The gel after running was fixed, stained, and washed according to the manufacturer's instructions. The density of brightness of stained gel was quantitatively analyzed by an iBright 1500 instrument (Thermo Fisher Scientific) and iBright Analysis Software ver 4.0.0 (Thermo Fisher Scientific).

#### Capillary gel electrophoresis

AAV samples for CGE measurement were prepared mostly according to the reference procedure as previously reported.^20^ The AAV solutions of volume 10 μL (∼5.8 × 10^10^ vg) were denatured and buffer exchanged following the protocol and the finally collected sample was diluted by 70 μL of deionized water for injection. CGE measurement was performed using a PA800Plus system (Sciex, Framingham, MA, USA). Prepared samples were injected with water plug sample stacking. Detection was performed at 214 nm using a PDA detector.

#### Peptide mapping analysis in solution

AAV samples were mixed with 50 mM ammonium bicarbonate (FUJIFILM Wako Chemicals, Osaka, Japan), 6 M guanidine hydrochloride (FUJIFILM Wako Chemicals), 3 mM tris (2-carboxyethyl) phosphine (FUJIFILM Wako Chemicals), and 3 mM iodoacetamide (FUJIFILM Wako Chemicals). The mixture was incubated at 37°C for 1 h in a dark place for denaturation, reducing, and alkylation. Then, the sample solution was desalted by buffer exchange into acetonitrile using MonoSpin C18 (GL Sciences, Inc., Tokyo, Japan). The collected samples were freeze dried by an Epsilon 2-4 LSCplus Freeze-Dryer (Martin Christ Gefriertrocknungsanlagen GmbH, Osterode, Germany). The collected pellet was dissolved in 10% acetonitrile in water and digested by trypsin (Promega, Madison, WI, USA) for 30 min at 37°C. Digested samples were injected into a nanoElute UHPLC (CTC Analytics AG, Zwingen, Switzerland) coupled to a trapped ion mobility spectrometer with time-of-flight instrument (Bruker, Billerica, MA, USA). Data analysis with ion mobility data was carried out by PEAKS software ver 10.6 (Bioinformatics Solutions, Inc., Waterloo, Canada).

#### LC-UV-MS with intact VPs

AAV samples were denatured with 10% acetic acid (FUJIFILM Wako Chemicals) and incubated at room temperature for 15 min. For the on-column denaturation experiment, AAV original solutions were directly used without acetic acid treatment. AAV samples were injected to Nexra HPLC (Shimadzu, Kyoto, Japan) coupled with a maXis II ETD ESI-QTOF mass spectrometer (Bruker). The separation was performed on an ACQUITY BEH C4 column (300 Å, 1.7 μm, 2.1 × 150 mm; Waters, Milford, MA, USA) at a flow rate of 0.2 mL/min and temperature of 80°C. Mobile phases A and B were 0.1% difluoroacetic acid (Waters) in MS grade water (Kanto Kagaku Co., Ltd., Tokyo, Japan) and in acetonitrile (Thermo Fisher Scientific), respectively. The pH values of solution A and mixed solution of 95% solution A and 5% solution B were measured as 1.93 and 1.90, respectively. Intact VPs were eluted from 32% B to 36% B gradient in 15 min. UV adsorption at 280 nm and intrinsic fluorescence of aromatic amino acid with setting of excitation wavelength at 280 nm and emission wavelength at 350 nm were utilized for detection of LC chromatogram. The deconvolution analysis was performed in the mass range of 40,000–90,000 Da to determine the mass values of VP components in the eluted peaks. Visualization and processing of mass spectra were performed by Bruker Compass DataAnalysis software ver 5.1 (Bruker) and the Maximum Entropy was used for deconvolution. PTM peaks were determined from both raw MS spectra and the deconvoluted mass spectra as detailed in Supplementary Fig. S3.

## Results

### Identification of VP variants by gel electrophoresis

**Figure 1.**
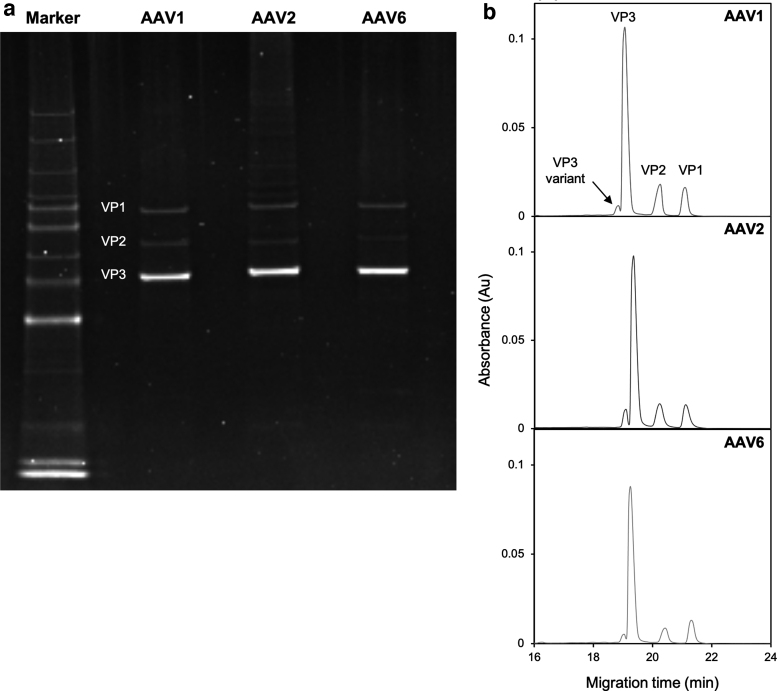
Evaluation of VP components by electrophoresis. **(a)** SDS-PAGE result of AAV1, AAV2, and AAV6. **(b)** CGE electropherograms of AAV1, AAV2, and AAV6. CGE, capillary gel electrophoresis; SDS-PAGE, sodium dodecyl sulfate–polyacrylamide gel electrophoresis; VP, viral protein.

SDS-PAGE is a conventional method used to evaluate the purity of VPs in the AAV capsid. In this study, SYPRO^®^ Ruby stain was used to detect even minor populations of AAV capsid protein. Stained gel of SDS-PAGE is shown in Fig. 1a. Three bands corresponding to VP1, VP2, and VP3 based on their molecular weight were detected in all AAV serotypes: AAV1, AAV2, and AAV6.

CGE measurement was then performed to evaluate the VP components. CGE gives better separation and sensitivity than conventional slab SDS-PAGE with SYPRO^®^ Ruby staining.^26^ Electropherograms of CGE measurement with detection at 214 nm are shown in Fig. 1b. In addition to VP1, VP2, and VP3 peaks, a minor peak before VP3 was additionally confirmed. This minor peak close to the VP3 position was reported in CGE results in previous studies,^20,26^ suggesting the existence of other VP components of AAV capsid. The estimated molecular weight of this minor peak from the calibration curve based on VP1, VP2, and VP3 is shown in Supplementary Fig. S1a. The unknown component contained in the minor peak was likely to have a lower molecular weight than VP3 by several thousand Daltons.

### Determination of VP3 variant sequence by mass spectrometry methods

**Figure 2.**
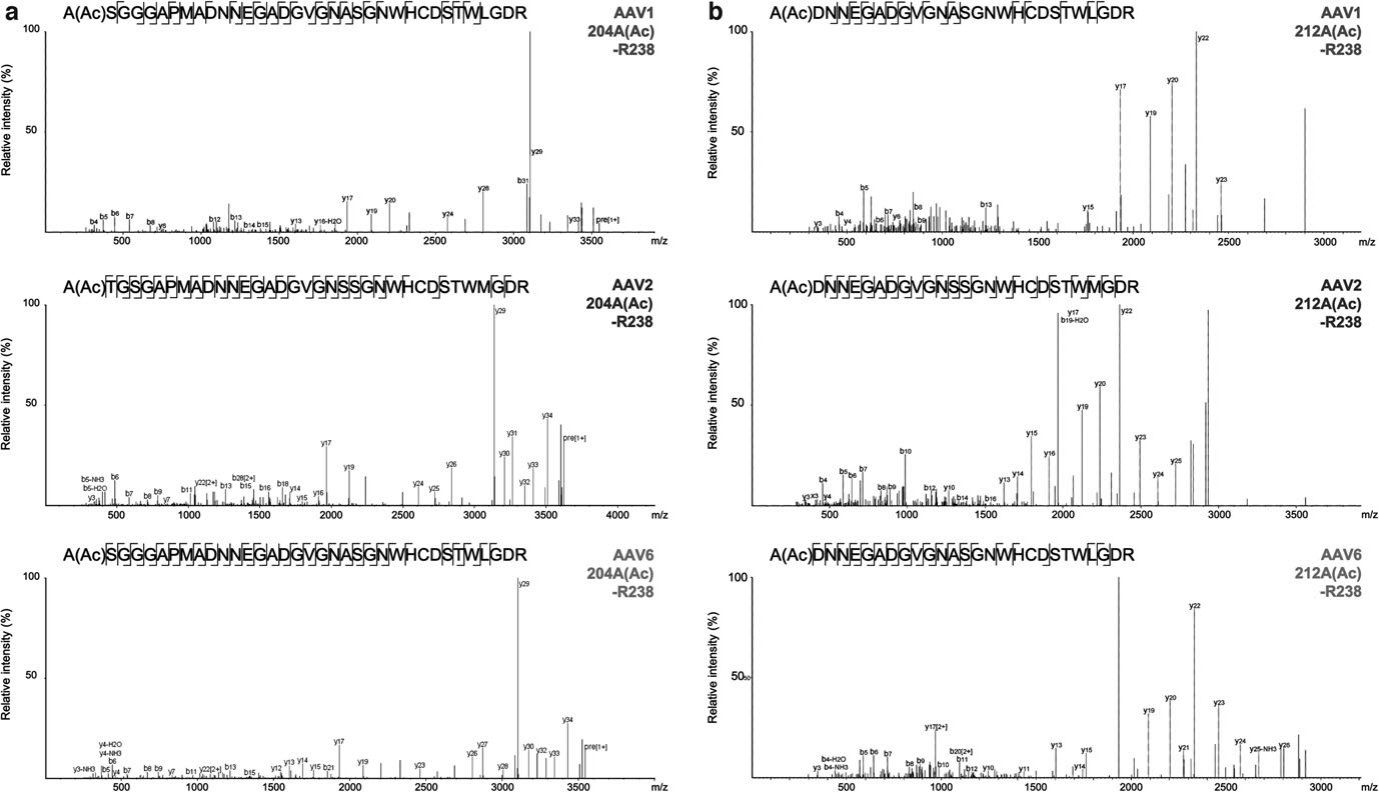
MS/MS spectra of N-terminal peptides detected by peptide mapping analysis. **(a)** VP3N-terminal peptide of which translation started at M203. **(b)** VP3 variant N-terminal peptide of which translation started at M211. MS, mass spectrometry.

Two types of mass spectrometry measurements were employed to determine the sequence of the unknown component in CGE measurement. First, peptide level analysis was conducted by in-solution peptide mapping by trypsin. It is well known that the N-terminal residue of VP3 is a highly acetylated Ala residue after excision of the Met residue.^24,28^ Meanwhile, two acetylated peptides were identified in AAV1, AAV2, and AAV6 as shown in Fig. 2. Among the assigned sequence number based on the VP1 sequence of a total of 736 residues of AAV1, one acetylated peptide for AAV1 is A(Ac)SGGGAPMADNNEGADGVGNASGNWHCDSTWLGDR, from A204 to the first cleavage site of trypsin R238 (Fig. 2a). Given that the theoretical sequence of VP3 starts at M203, it was suggested that the excision of the N-terminal methionine residue on M203 and the acetylation of the next alanine residue on A204 were processed during the expression of VP3 in the host cell. Thus, this peptide was surely derived from the N-terminal regions of VP3.

Another acetylated peptide is A(Ac)DNNEGADGVGNASGNWHCDSTWLGDR, from A212 to R238 (Fig. 2b). We focused on the fact that the amino acid residue at position 211 is also a methionine residue, close to the N-terminal methionine residue of VP3, M203. Besides, acetylation is one of the most common PTMs after N-terminal Met excision in eukaryotes, not generated from a sample preparation step. Therefore, the acetylated peptide A212(Ac)-R238 indicated the existence of the VP3 variant of which the translation started at M211.

**Figure 3.**
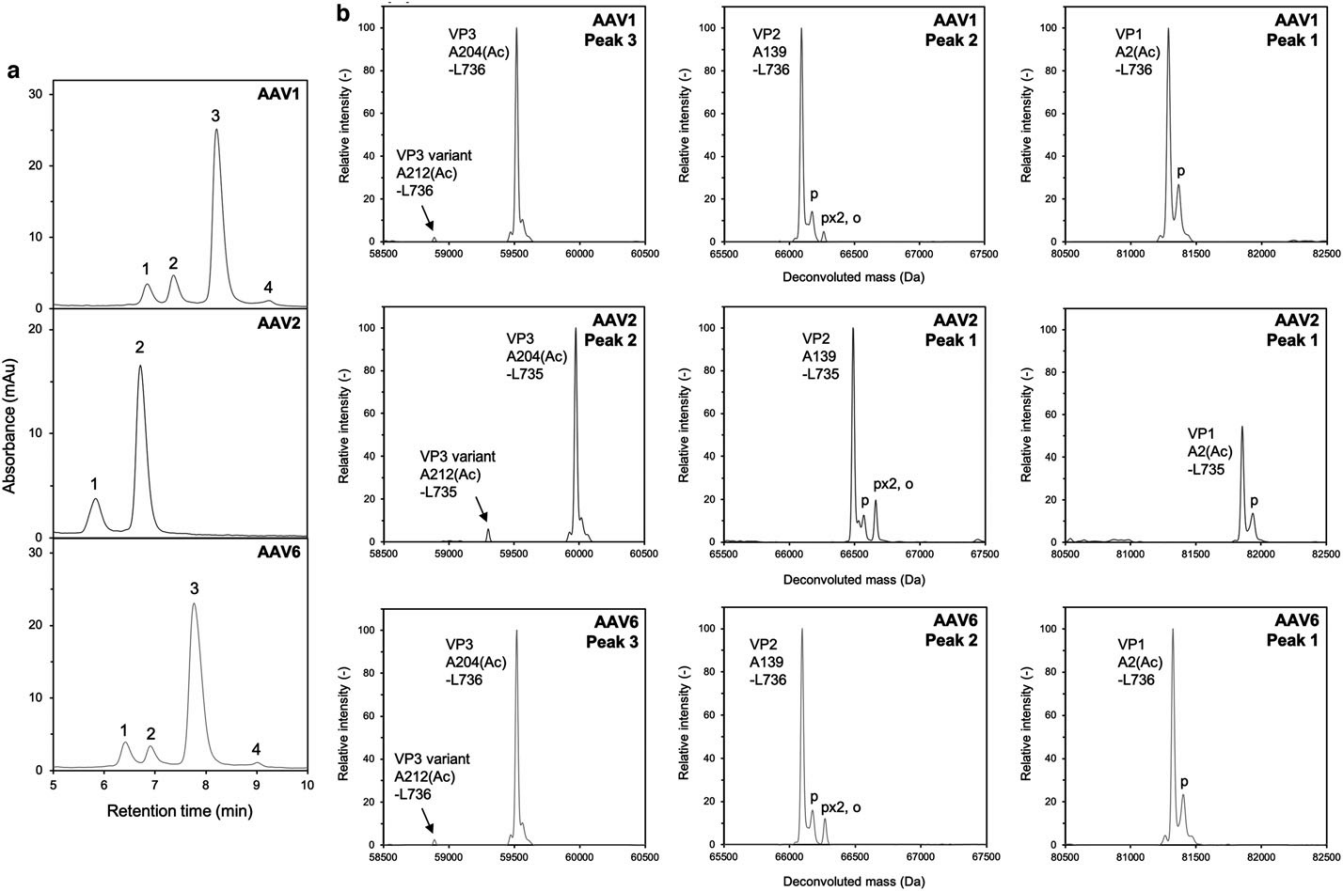
Detection of VP1, VP2, VP3, and VP3 variant by LC-UV-MS measurement. **(a)** LC chromatograms of AAV1, AAV2, and AAV6. **(b)** Deconvoluted mass spectra of separated peaks (Peaks 1, 2, and 3 in AAV1 and AAV6, and peaks 1 and 2 in AAV2). The peak number shown at the *upper right* corresponds to the number depicted in Fig. 3a. The label “p” denotes the phosphorylation and “o” oxidation. LC-UV-MS, liquid chromatography–UV–mass spectrometry.

Second, LC-UV-MS was performed to determine VP components at intact protein level. The LC chromatogram with UV detection at 280 nm is shown in Fig. 3a. Denatured VPs were separated into four peaks for AAV1 and AAV6, and two peaks for AAV2. Deconvolution analysis of the eluted peaks was performed as described in Experimental section. The deconvoluted mass values of intact VPs are listed in Table 1. No components were confirmed other than the VPs and VP-related components listed in Table 1, as shown in the overall deconvoluted mass spectra (Supplementary Fig. S2). VP1, VP2, and VP3 were individually eluted as different peaks in AAV1 and AAV6, whereas VP1 and VP2 were coeluted as a single peak in AAV2. In all serotypes, the main peaks of VP1, VP2, and VP3 without modification were detected within 30 ppm mass error (<2.5 Da) compared with the theoretical values. The deconvoluted mass spectra focusing on the VP peaks of all serotypes are shown in Fig. 3b.

**Table 1.** *Deconvoluted mass list detected in liquid chromatography*–*UV*–*mass spectrometry*

Serotype	Peak no.	Tentative identities	Predicted amino acid sequence	Actual amino acid sequence	Measured mass (Da)	Theoretical mass (Da)	Mass accuracy (ppm)
AAV1	1	VP1	M1-L736	A2(Ac)-L736	81288.2	81285.9	29.0
	2	VP2	T138-L736	A139-L736	66094.6	66093.1	22.4
	3	VP3	M203-L736	A204(Ac)-L736	59517.1	59516.9	4.4
		VP3 variant	M211-L736	A212(Ac)-L736	58888.0	58888.2	3.7
	4	VP3 fragment	M203-D626	A204(Ac)-D626	47197.9	47197.1	18.5
		VP3 fragment	M203-D590	A204(Ac)-D590	43271.0	43270.6	10.1
AAV2	1	VP1	M1-L735	A2(Ac)-L735L	81857.3	81855.3	24.8
		VP2	T138-L735	A139-L735	66489.8	66488.2	24.1
	2	VP3	M203-L735	A204(Ac)-L735	59974.5	59974.0	8.2
		VP3 variant	M211-L735	A212(Ac)-L735	59302.2	59301.3	14.7
AAV6	1	VP1	M1-L736	A2(Ac)-L736	81324.2	81322.0	26.8
	2	VP2	T138-L736	A139-L736	66096.9	66095.2	25.8
	3	VP3	M203-L736	A204(Ac)-L736	59519.4	59519.0	7.0
		VP3 variant	M211-L736	A212(Ac)-L736	58890.6	58890.3	5.2
	4	VP3 fragment	M203-D626	A204(Ac)-D626	47177.0	47176.1	18.7
		VP3 fragment	M203-D590	A204(Ac)-D590	43222.1	43221.6	10.4

The peak number corresponds to the number in Fig. 3a.

Multiple populations of deconvoluted spectra indicated the PTM level of VPs. As shown in Table 2, phosphorylated VP1, phosphorylated VP2, and doubly-phosphorylated and oxidized VP2 were detected, whereas no PTM peaks were assigned for VP3. Although the monophosphorylation level of VP1 and VP2 was similar across AAV serotypes, the population of VP2 with two phosphorylations and oxidation was different. In the most abundant peaks in LC (peak no. 3 for AAV1 and AAV6, no. 2 for AAV2 in Fig. 3a), the small populations besides VP3 were detected as shown in Fig. 3b (left panels). The small population had a slightly lower mass value than VP3 and matched the sequences as A212(Ac)-L736 for AAV1 and AAV6, and A212(Ac)-L735 for AAV2. The N-terminal region of the small population was consistent with the second acetylated peptide detected in peptide mapping results (Fig. 2b), suggesting that the translation of VP3 also started from the second methionine residue, M211 to the C-terminal residue. Taken together, the existence of VP3 variant which had about 10 kDa lower molecular weight than VP3 was confirmed by both peptide mapping and LC-UV-MS measurements. Based on the molecular weight, the VP3 variant corresponded to a minor peak in CGE measurement.

**Table 2.** Evaluation of post-translational modification level of viral proteins

Serotype	Peak no.	Tentative identities	Measured mass (Da)	Theoretical mass (Da)	Mass shift (Da)	Possible PTM	Relative peak ratio (%)
AAV1	1	VP1	81288.2	81285.9	—	—	100.0
			81364.9	81365.8	76.7	Phosphorylation	26.8
	2	VP2	66094.6	66093.1	—	—	100.0
			66173.3	66173.1	78.7	Phosphorylation	14.3
			66265.2	66269.1	170.6	Phosphorylation × 2, Oxidation	4.9
AAV2	1	VP1	81857.3	81855.3	—	—	100.0
			81935.6	81935.2	78.3	Phosphorylation	25.2
		VP2	66489.8	66488.2	—	—	100.0
			66568.8	66568.2	79.0	Phosphorylation	12.6
			66662.0	66664.2	172.2	Phosphorylation × 2, Oxidation	19.8
AAV6	1	VP1	81324.2	81322.0	—	—	100.0
			81401.8	81402.0	77.6	Phosphorylation	23.4
	2	VP2	66096.9	66095.2	—	—	100.0
			66173.9	66175.2	77.0	Phosphorylation	16.0
			66270.4	66271.2	173.5	Phosphorylation × 2, Oxidation	12.1

Relative peak ratio is calculated from the intensity of deconvoluted peaks in Fig. 3b. The intensity of the unmodified peak in each VP is set as 100.0%.

PTM, post-translational modification; VP, viral protein.

### Degradation of VPs under the conditions of low pH and high temperature

**Figure 4.**
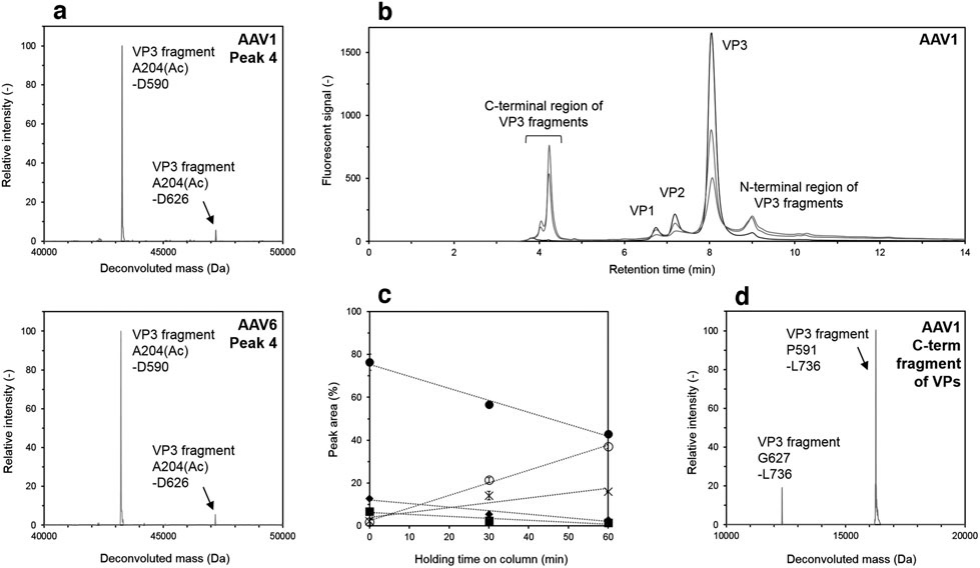
Detection of VP3 fragments and evaluation of the effect of column holding time on the generation of VP3 fragments. **(a)** Deconvoluted mass spectra of peak 4 in AAV1 and AAV6. The peak number shown at the *upper right* corresponds to the number depicted in Fig. 3a. **(b)** LC chromatogram of AAV1 samples treated with different column holding time. The *black*, *blue*, and *red lines* represent the column holding time of 0, 30, and 60 min, respectively. **(c)** Traces of the peak area of VP components and fragments. The peak area is calculated from (**b**). *Closed circles*, *diamonds*, and *squares* correspond to VP3, VP2, and VP1, whereas cross marks and *open circles* represent the N-terminal region of VP3 fragments and C-terminal region of VP3 fragments, respectively. **(d)** Deconvoluted mass min in spectra of the peak eluted at around 4 min in (**b)**. Deconvolution analysis was performed with the data of AAV1 treated with the column holding time of 60 min.

In the LC chromatogram of AAV1 and AAV6, a fourth peak that could not be assigned to conventional VPs was detected. The deconvoluted mass spectra of peak 4 in the LC chromatogram of AAV1 and AAV6 are shown in Fig. 4a. Two types of VP3 fragment, A204(Ac)-D590 and A204(Ac)-D626 were detected. The determined sequence of VP3 was A204(Ac)-L736, therefore, it was indicated that cleavage occurred at the specific sequence sites between D590 and P591, and between D626 and G627. The molecular weight of the VP3 fragments were about 47.2 and 43.4 kDa, however, there were no bands or peaks corresponding to those in SDS-PAGE or CGE based on the molecular weight. Previous studies indicated that the cleavage at the DP or DG sequence frequently occurred under acidic pH conditions using hexapeptide or antibody,^29^ and the clipping VPs of AAV were detected in several AAV serotypes.^27,30^ Considering that these VP3 fragments were not detected by SDS-PAGE and CGE, the LC system with low pH of the mobile phase and high temperature of the column oven may be the cause of the specific cleavages.

To verify this hypothesis, AAV samples after injection were treated at low pH (Mobile phase B 5%) and high temperature (Column oven: 80°C) in the chromatography column. Five percent mobile phase B was the initial condition of the LC gradient in this study, which means the mixture of 95% mobile phase A (0.1% difluoroacetic acid [DFA] in MS grade water) and 5% mobile phase B (0.1% DFA in MS grade acetonitrile). LC chromatograms for various column holding times of the sample on the chromatography column are shown in Fig. 4b. Injected samples were exposed to 5% mobile phase B (around pH 2.0) in the column incubated at 80°C during the column holding time. The black line corresponds to the elution profile in which the LC gradient started immediately after the injection, and the blue and red lines represent the profiles in which the AAV samples were eluted after the column holding time of 30 or 60 min before starting the LC gradient. As the column holding time increased, the peak area of VP3 fragments at 9 min also increased (Fig. 4c), indicating that the cleavage at D590-P591 and D626-G627 was facilitated in the chromatography column.

Simultaneously, an additional peak at the retention time of around 4 min emerged. Deconvolution analysis revealed that this additional peak matched the C-terminal regions of VP fragments (P591-L736 and G627-L736) as shown in Fig. 4d. The VP1 and VP2 contain the whole sequence of VP3, however, N-terminal regions of VP1 and VP2 could not be detected probably due to the small amounts of VP1 and VP2. Taken together, these results indicated that the populations of VP1, VP2, and VP3 decreased due to the increasing amount of VP3 fragments with column holding time.

### Determination of VP ratio by SDS-PAGE, CGE, and LC-UV-MS

The VP ratio of the AAV capsid was determined by calculation from the brightness density of the bands in SDS-PAGE, the peak area of the electropherogram in CGE, and the peak area of the LC-UV chromatogram in LC-UV-MS. Since it is necessary to correct the experimental values to compare the molar VP ratios determined from the above methods, which are based on different principles, the obtained data were corrected as follows: the densities in SDS-PAGE were divided by the sum of the amino acid residues; K, R, H, Y, and W in VPs, which are suggested to be interaction residues with SYPRO^®^ Ruby dye^31^; the peak areas with detection at 214 nm in CGE were divided by the molar extinction efficient of VPs at 214 nm, which reflects the UV absorbance of peptide bonds and amino acids^32^; and the peak areas with detection at 280 nm in LC-UV-MS were divided by the molar extinction coefficient of VPs at 280 nm under denatured conditions.^33^ The first Met deletion of VP1 and VP3 determined from LC-UV-MS was taken into account in the calculation of the molar extinction coefficient. In addition, VP ratio was determined from the peak height of deconvoluted mass spectra.

Determined VP ratios by the four methods, SDS-PAGE, CGE, LC-UV-MS (LC-UV), and LC-UV-MS (MS-Deconvolution) are listed in Table 3. Among the determined VP ratios, CGE and LC-UV-MS (LC-UV) show relatively close values, except AAV2 with each other. VP2 ratios tend to be lower in SDS-PAGE, whereas VP3 ratios tend to be higher in LC-UV-MS (LC-UV) and LC-UV-MS (MS-Deconvolution). According to the VP ratio determined by CGE, in comparison with the proposed VP ratio 1:1:10, the ratio of VP3 tended to be lower than 10 in all serotypes, and the ratio of VP2 was higher than 1 in AAV1 and AAV2, whereas lower than 1 in AAV6.

**Table 3.** *Viral protein ratios determined by sodium dodecyl sulfate*–*polyacrylamide gel electrophoresis, capillary gel electrophoresis, and liquid chromatography*–*UV*–*mass spectrometry*

Serotype	Method	Detection	VP1	VP2	VP3	VP3 variant	VP3 fragments
AAV1	SDS-PAGE	SYPRO^®^ Ruby	1.0	0.5	9.3	ND^*^	ND
	CGE	UV 214 nm	1.0	1.4	8.2	0.5	ND
	LC-UV-MS (LC-UV)	UV 280 nm	1.0	1.8	13.7	ND^*^	0.5
	LC-UV-MS (MS-Deconvolution)	MS	1.0	1.7	12.0	0.2	0.9
AAV2	SDS-PAGE	SYPRO^®^ Ruby	1.0	0.4	11.6	ND^*^	ND
	CGE	UV 214 nm	1.0	1.4	7.6	0.6	ND
	LC-UV-MS (LC-UV)	UV 280 nm	VP1+VP2		10.5	ND^*^	ND
			2.0				
	LC-UV-MS (MS-Deconvolution)	MS	1.0	1.8	19.7	1.2	ND
AAV6	SDS-PAGE	SYPRO^®^ Ruby	1.0	0.3	9.2	ND^*^	ND
	CGE	UV 214 nm	1.0	0.8	8.1	0.3	ND
	LC-UV-MS (LC-UV)	UV 280 nm	1.0	1.0	11.9	ND^*^	0.2
	LC-UV-MS (MS-Deconvolution)	MS	1.0	0.7	8.3	0.2	0.6

The value for VP1 obtained by each method is set as 1.0. For AAV2 of LC-UV-MS (LC-UV), the value of VP1+VP2 is set as 2.0 because of the coelution of VP1 and VP2. LC-UV-MS (LC-UV) shows the ratio calculated from the peak area in LC chromatogram, and LC-UV-MS (MS-Deconvolution) shows the ratio calculated from the peak height of main peak in deconvoluted mass spectra. All values are the average of three replicates. ND denotes “not determined” and ND^*^ denotes “not determined because of poor separation.”

CGE, capillary gel electrophoresis; LC-UV-MS, liquid chromatography–UV–mass spectrometry; SDS-PAGE, sodium dodecyl sulfate–polyacrylamide gel electrophoresis.

## Discussion

Recent studies indicated the existence of an additional VP component.^20,24,26^ In this study, the VP3 variant of which the translation started at M211, not at M203, was identified by CGE and LC-UV-MS, and the VP3 fragments of which the C-terminal region was cleaved off between the DP or DG sequence were detected by LC-UV-MS.

With regard to the generation of the VP3 variant, the regulation of the expression level of VPs should be considered. In expression of the AAV Cap gene, alternative splicing and leaky scanning mechanisms for the synthesis of multiple proteins from a single pre-mRNA likely control the ratio of VPs. Previous studies indicated that the alternative splicing of Cap pre-mRNA resulted in the production of two spliced species: major spliced mRNA of 2.3 kb and minor spliced mRNA of 2.6 kb.^34,35^ The ratio of minor and major spliced products is roughly 1:7, while spliced population of pre-mRNA is changed as time after infection.^36,37^ Only the minor spliced mRNA has the initiation codon to translate VP1, thus the amount of VP1 is relatively low. Both VP2 and VP3 are produced from the same major spliced mRNA by the leaky scanning mechanism. The abundance of VP2 and VP3 is likely determined by their initiation codon: VP2 is translated from an unusual one, ACG, whereas VP3 starts from ATG, contributing to the lower expression ratio of VP2.^38^ These regulations underlie the proposed molar VP ratio of VP1:VP2:VP3 = 1:1:10.

**Figure 5.**
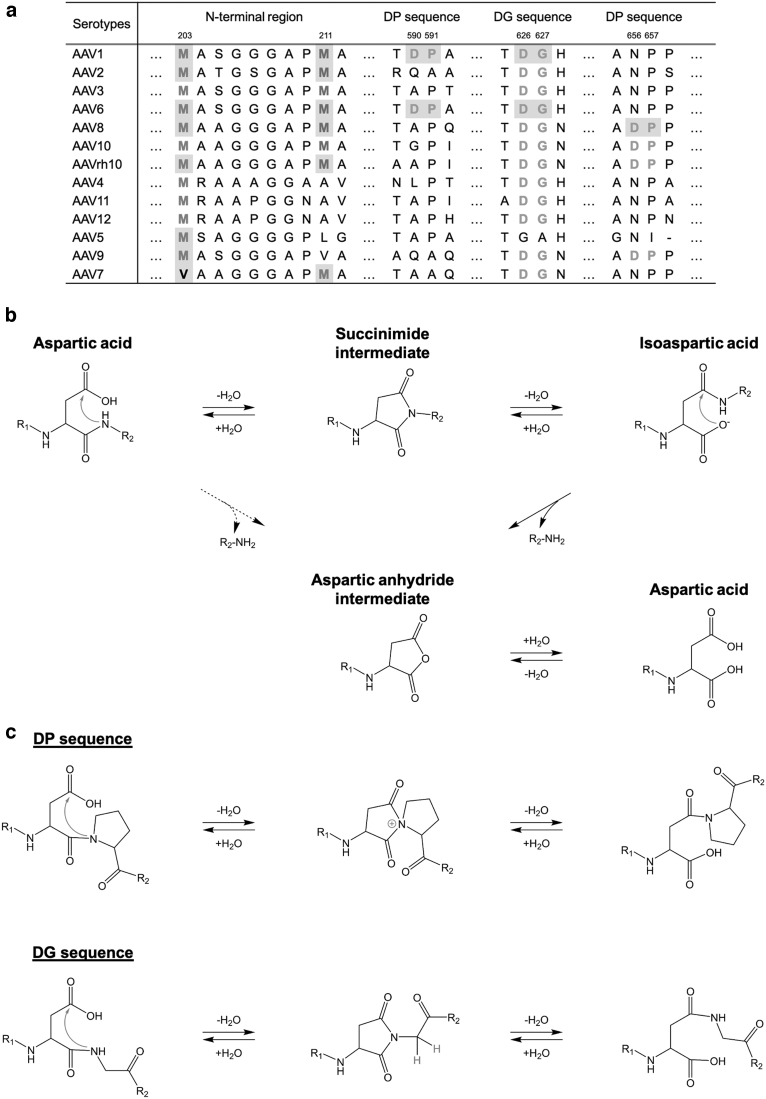
Sequence alignment of VP3 and reaction scheme of DP and DG fragmentation. **(a)** Sequence alignment of VP3 of AAV1-12 and AAVrh10 focusing on the N-terminal regions and cleavage regions at the DP or DG sequence. Residue number was assigned based on the VP1 sequence of AAV1. The VP3 variant and VP3 fragment experimentally confirmed in this study and previous studies^21,47^ are shown in *gray*. **(b)** Reaction schemes of isomerization of an aspartic acid residue and hydrolysis of DX sequence. **(c)** Isomerization reactions of DP and DG sequence.

In this study, the VP3 variant (M211-L736 for AAV1 and AAV6, M211-L735 for AAV2) was detected both at the peptide and intact protein level. Since even three or four distinct proteins are translated from a single transcript by leaky scanning,^39^ it is possible that a portion of ribosomal subunits reaches the second initiation codon of VP3 at M211 after missing the noncanonical initiation codon of VP2 and the initiation codon of VP3. As discussed in a previous study,^24^ the Kozak sequence may control the expression level of VP3 and the VP3 variant. If there are two possible initial codons to translate, the initiation codon, which has A in the −3 position besides G in the +4 position (A in the initiation codon AUG is counted as +1), is favorable in the expression level.^40,41^ In the case of VP3 of some AAV serotypes, the first ATG at M204 has A in −3 and G in +4, whereas the second ATG at M211 has C in −3 and G in +4 (Supplementary Fig. S4a). Therefore, the population of the VP3 is much larger compared with the VP3 variant. The sequence alignment of N-terminal regions of VP3 is shown in Fig. 5a. Multiple AAV serotypes (AAV1, AAV2, AAV3, AAV6, AAV8, AAV10, and AAVrh10) have two potential initiation sites. The existence of the VP3 variant was experimentally confirmed for AAV1, AAV2, and AAVrh10 by intact-MS in a previous study,^24^ and for AAV1, AAV2, and AAV6 by LC-UV-MS and peptide mapping in this study. At least for AAV1, AAV2, AAV6, and AAVrh10, the VP3 variant is possibly contained in AAV vector.

We also clearly showed that the VP3 variant was separated as a minor peak just before VP3 in CGE measurement. Both in LC-UV-MS and CGE, the VP3 variant was also detected as a minor peak compared with VP3. Consistently, AAV5, which had only one initiation codon of VP3 showed no additional peak in CGE (Supplementary Fig. S4b). The VP3 variant should be quality controlled to guarantee the purity of AAV products for human gene therapy, although further research is required to investigate the biological function of the VP3 variant.

The specific sequences of VP3 could be cleaved under the analysis condition of low pH (pH 2.0) and high temperature (80°C), resulting in the generation of VP3 fragments. In LC-MS measurement with intact VP samples, it is required to separate VP peaks in LC to obtain well-resolved MS spectra and accurate deconvoluted mass value.^24,27,30^ Since the denatured VPs were hardly separated at room temperature of 25°C (Supplementary Fig. S5a), the high temperature of 80°C was applied to the chromatography column to improve the separation of VP peaks of AAV samples in LC. The addition of an ion-pairing reagent, DFA 0.1% to the mobile phase resulting in pH around 2.0, is also essential for better separation instead of formic acid.^27,30,42^ We also prepared the synthesized peptide, which had the same amino acid sequence of AAV1 VP3 containing the DP sequence, and confirmed that DP sequence was specifically cleaved (Supplementary Fig. S5b). Thus, high temperature and low pH are imperative for well-resolved VP separation. However, the drawback of these conditions is VP3 fragmentation, in which the C-terminal region is cut off at the specific sites of the DP and DG sequence in AAV1 and AAV6.

The amino acid sequence DX, an aspartic acid and arbitrary amino acid residues, respectively, is susceptible to cleaving due to hydrolysis under acidic conditions.^29^ To explain the fragmentation mechanism at specific sites in this study, the reaction pathway of the isomerization and hydrolysis at the DX sequence is shown in Fig. 5b. Aspartic acid residues in proteins can transform into isoaspartic acid through succinimide intermediate. Aspartic acid has a β-carboxyl group, whereas isoaspartic acid has a α-carboxyl group with pKa value of 0.8–1.7 U lower than that of β-carboxyl group.^43^

Even if the pKa of carboxyl group can vary due to the influence of neighboring residues, given that the value of pK1 (α-carboxyl group) is 1.88 and that of pK2 (β-carboxyl group) is 3.65 for aspartic acid,^44^ it is likely that the α-carboxyl group of isoaspartic acid is mainly ionized but the β-carboxyl group of aspartic acid is rarely ionized under acidic condition of around pH 2.0. The ionized α-carboxyl group is a trigger for the formation of the reactive intermediate containing aspartic anhydride by nucleophilic attack. Then, N-terminal peptide is released and the reactive intermediate is subsequently hydrolyzed to an aspartic acid residue. In general, the DP or DG sequence is frequently cleaved among DX sequences.^29^ As suggested previously, the basic nature of the nitrogen in a proline residue or the steric hindrance of side chain in a glycine residue facilitates the isomerization of an aspartic acid residue (Fig. 5c).^45,46^ Moreover, the succinylation reaction of an aspartic acid residue is also accelerated at high temperature,^47,48^ whereas DP peptide showed considerable cleavage but was independent of Arrhenius temperature.^46^

In this study, only LC-UV-MS includes the process of exposing the AAV samples to the conditions of low pH of around 2.0 and high temperature of 80°C. Therefore, it is suggested that VP3 fragments are the only products in LC-UV-MS but are not generated in analysis procedures of SDS-PAGE or CGE in this study. Although LC-UV-MS has the drawback of DP and DG cleavage, the population of VP3 fragments is small at <3% of total peak area in the LC chromatogram when the gradient is immediately started after sample injection. Even in such immediate elution, injected AAV samples are held on the chromatography column for about 8 min until the elution of VP3 for AAV1 and AAV6. Since the slope of the generation of VP3 fragments is 0.23% per minutes of the column holding time in Fig. 4c, it is indicated that most of the AAV samples before measurement are not cleaved at the DP or DG sequence. Cleavage % of DP sequence in synthesized peptide is 6.1%, suggesting that the folding of DP sequence in VP3 suppress the cleavage. Therefore, we conclude that the small amount of VP3 fragments less than 3% should be included in the quantification of VP3. As discussed above, one of the causes of the VP3 fragmentation is low pH. During the manufacturing of AAV vector, AAV vector can be exposed to an acidic pH in the purification step using affinity chromatography.^49^ Then, to confirm whether DP or DG cleavage occurred during affinity chromatography, AAV1 samples were formulated in the elution buffer (100 mM sodium citrate buffer with 100 mM NaCl and 0.001% [w/v] poloxamer at pH 3.5), of which the buffer composition was the same as that used in affinity chromatography.^49^ After elution, purified AAV samples are likely preserved under refrigerated conditions until buffer exchange. Therefore, AAV1 samples in the elution buffer were incubated at 4°C and LC-UV-MS was performed for different times. The amount of VP3 fragments did not exceed 3%, the imperative amount produced by LC-UV-MS, indicating that the DP or DG cleavage was not facilitated in the elution buffer at pH 3.5 until 72 h (Supplementary Fig. S5c). This result supports that VP3 fragment is only produced under the analytical conditions in LC-UV-MS.

It has been proposed that the AAV capsid is composed of three VPs: VP1, VP2, and VP3, which assemble in the ratio of ∼1:1:10. This stoichiometry was estimated based on the analysis of VP components by electrophoresis.^4,5^ However, recent studies indicated the heterogeneity of the VP stoichiometry.^50,51^ It should be noted that the unbalanced stoichiometry might originate from the recombinant production of AAV. Even if VP ratio of AAV vector is heterogeneous or varies depending on producing method, determination of bulk VP ratio is still much required for quality control of AAV vector. Well-resolved separations are essential to quantify the amounts of VPs, including the VP3 variant. In the SDS-PAGE measurement, while the additional band just under the VP3 was found in a few previous studies, including reference standard material of AAV,^18,52^ the bands of VP3 and VP3 variant were overlapped in this study. This fact suggested that the amount of VP3 variant varied among AAV samples. The VP ratio determined from SDS-PAGE with fluorescent dye was different from that of CGE or LC-UV-MS (LC-UV) because of the ungrasped relations of binding amounts of dye to VPs. In addition to the quantification of VPs, the amino acid length of all the VP components could be unambiguously identified by LC-UV-MS. As shown in Table 3, the VP ratios of VP1 and VP2 estimated from LC-UV-MS (LC-UV) approximately match the ratios determined by CGE in AAV1 and AAV6, of which VP1 and VP2 can be separated in LC. The overestimation of VP3 ratios in LC-UV-MS (LC-UV) might be due to the fact that the peak areas of VP3 peaks in LC-UV chromatogram contained both VP3 and VP3 variants. On the other hand, the VP ratios in LC-UV-MS (MS-Deconvolution) was different from that of LC-UV-MS (LC-UV) or CGE. One of the possible causes of this discrepancy is the difference of ionization efficiency between the amino acid sequence of VPs.

From these points, it seems better to employ the CGE method for the quantification of VPs because all peaks had only one VP component. While the VP3 variant can be separated as a minor peak resulting in the appropriate quantification of VPs, the actual sequence of VPs cannot be determined. In conclusion, combining the identification of VP components by LC-UV-MS into the peak assignment in CGE results is necessary for accurate and quantitative analysis of VP components derived from AAV vectors.

## Conclusion

In this study, SDS-PAGE, CGE, peptide mapping, and LC-UV-MS were carried out for the characterization of VP components of AAV1, AAV2, and AAV6. First, in all serotypes, the VP3 variant of which the translation started at M211, not at M203, was identified by peptide mapping and LC-UV-MS. The VP3 variant could be generated by leaky scanning at the first initial codon of VP3. Likewise, AAV3, AAV8, AAV10, and AAVrh10 could have two potential initiation codons. We also indicated that the VP3 variant was separated as a minor peak just before VP3 in CGE measurement. Second, in AAV1 and AAV6, the VP3 fragments of which the C-terminal regions ended at D590 or D626 were detected by LC-UV-MS. The driving force of the cleavage at the DP and DG sequence is the hydrolysis reaction through the reactive intermediate of aspartic anhydride, which was facilitated in the chromatography column under the conditions of low pH (around pH 2.0) and high temperature (80°C). VP3 fragment was only produced during the LC-UV-MS analysis and accounted for only small population with <3% of the total peak area. Thus, the VP3 fragments should be included in the quantification of VP3. Finally, the analytical methods for evaluating VP components employed in this study were compared. Complementarity analysis using CGE and LC-UV-MS is effective for the comprehensive assessment of the VP purity of AAV vectors.

## Supplementary Material

Supplemental data

Supplemental data

Supplemental data

Supplemental data

Supplemental data
