# Supplementary material for: Characterization of Adeno-Associated Virus Capsid Proteins with Two Types of VP3-Related Components by Capillary Gel Electrophoresis and Mass Spectrometry
Source: Hum Gene Ther. 2021 Nov 15;32(21-22):1403–16. doi: 10.1089/hum.2021.009 (PMC10112878; doi:10.1089/hum.2021.009)
Supplement: Supplemental data [file Suppl_FigureS3.docx]

**Figure S3.** PTM detection by the raw mass spectra and the deconvoluted mass spectra. Results of AAV1 was shown as representative for comparison.


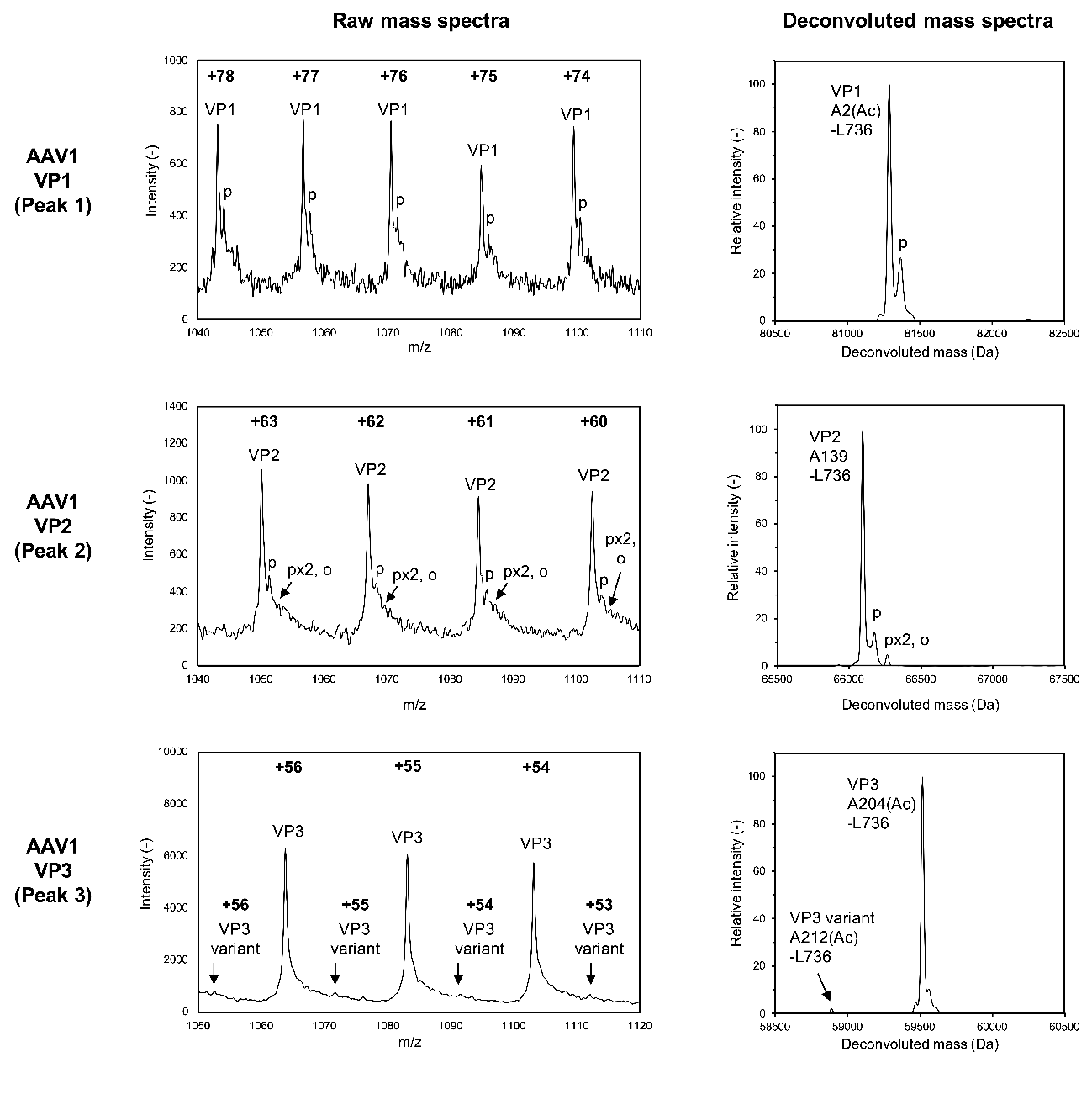


The PTM peaks were evaluated both in the raw MS spectra and the deconvoluted mass spectra. For VP1 and VP2, PTM peaks could be confirmed in the raw MS spectra in addition to the deconvoluted mass spectra. Focusing on the VP3, while it looks that several shoulder peaks were emerged near the main peak in the deconvoluted mass spectra, no PTM peaks were detected in the raw MS data. Thus, the shoulder peaks near the main peak of VP3 in the deconvoluted mass spectra could be the artificial ones produced by deconvolution analysis. The same is true for VPs of AAV2 and AAV6.
