# Supplementary material for: Characterization of Adeno-Associated Virus Capsid Proteins with Two Types of VP3-Related Components by Capillary Gel Electrophoresis and Mass Spectrometry
Source: Hum Gene Ther. 2021 Nov 15;32(21-22):1403–16. doi: 10.1089/hum.2021.009 (PMC10112878; doi:10.1089/hum.2021.009)

**Figure S1.** Calibration curves and estimated values of VP molecular weight.

(a) Calibration curve based on the migration time of VP1, VP2, and VP3.

The calibration curve was fitted linearly because only three data points were available for calibration and the data point of the VP3 variant was out of the range of the calibration curve. The estimated molecular weight of the VP3 variant was several thousand lower than that of VP3.

(b) Calibration curve based on the migration time of molecular weight marker (SDS-MW Size Standard (10 kDa to 225 kDa, 16 mg/mL), BECKMAN COULTER, Brea, CA).

The calibration curve was fitted linearly with the logarithm of molecular weight since the data points of VP1, VP2, VP3, and VP3 variant were located within the calibration range. Overestimation of the molecular weight of VP components was possibly due to the difference of the buffer excipient between the molecular weight marker and AAV samples.


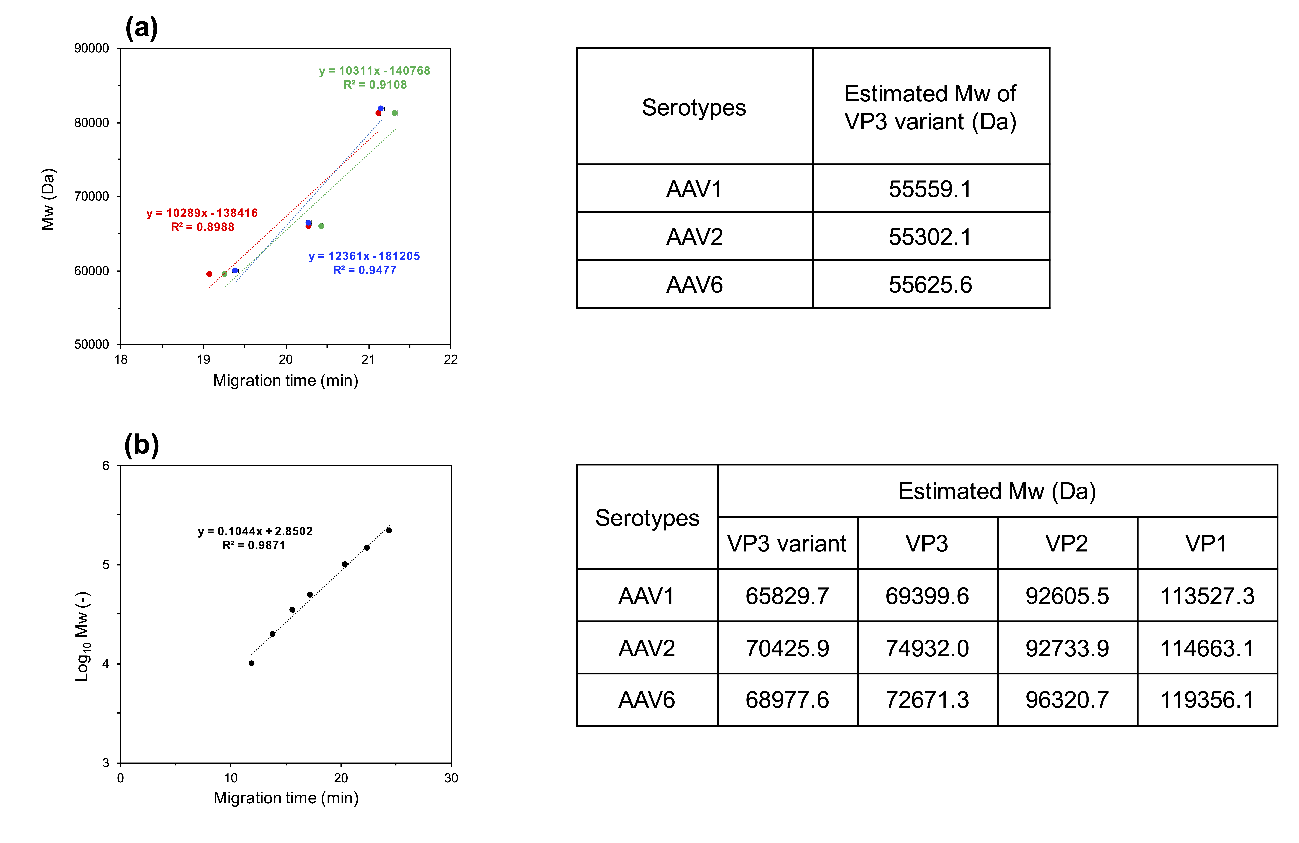

Supplement: Supplemental data [file Suppl_FigureS1.docx]
