# Supplementary material for: Characterization of Adeno-Associated Virus Capsid Proteins with Two Types of VP3-Related Components by Capillary Gel Electrophoresis and Mass Spectrometry
Source: Hum Gene Ther. 2021 Nov 15;32(21-22):1403–16. doi: 10.1089/hum.2021.009 (PMC10112878; doi:10.1089/hum.2021.009)

**Figure S2.** Overviews of deconvoluted mass spectra in the range of 40000–90000 Da.

For AAV1 and AAV6, red, blue, green, and black lines represent the deconvoluted mass spectra of peak 1 (VP1), peak 2 (VP2), peak 3 (VP3 and VP3 variant), and peak 4 (VP3 fragment), respectively. For AAV2, the red and green lines represent the deconvoluted mass spectra of peak 1 (VP1 and VP2) and peak 2 (VP3 and VP3 variant), respectively. The peak number corresponds to the number depicted in Figure 3a.


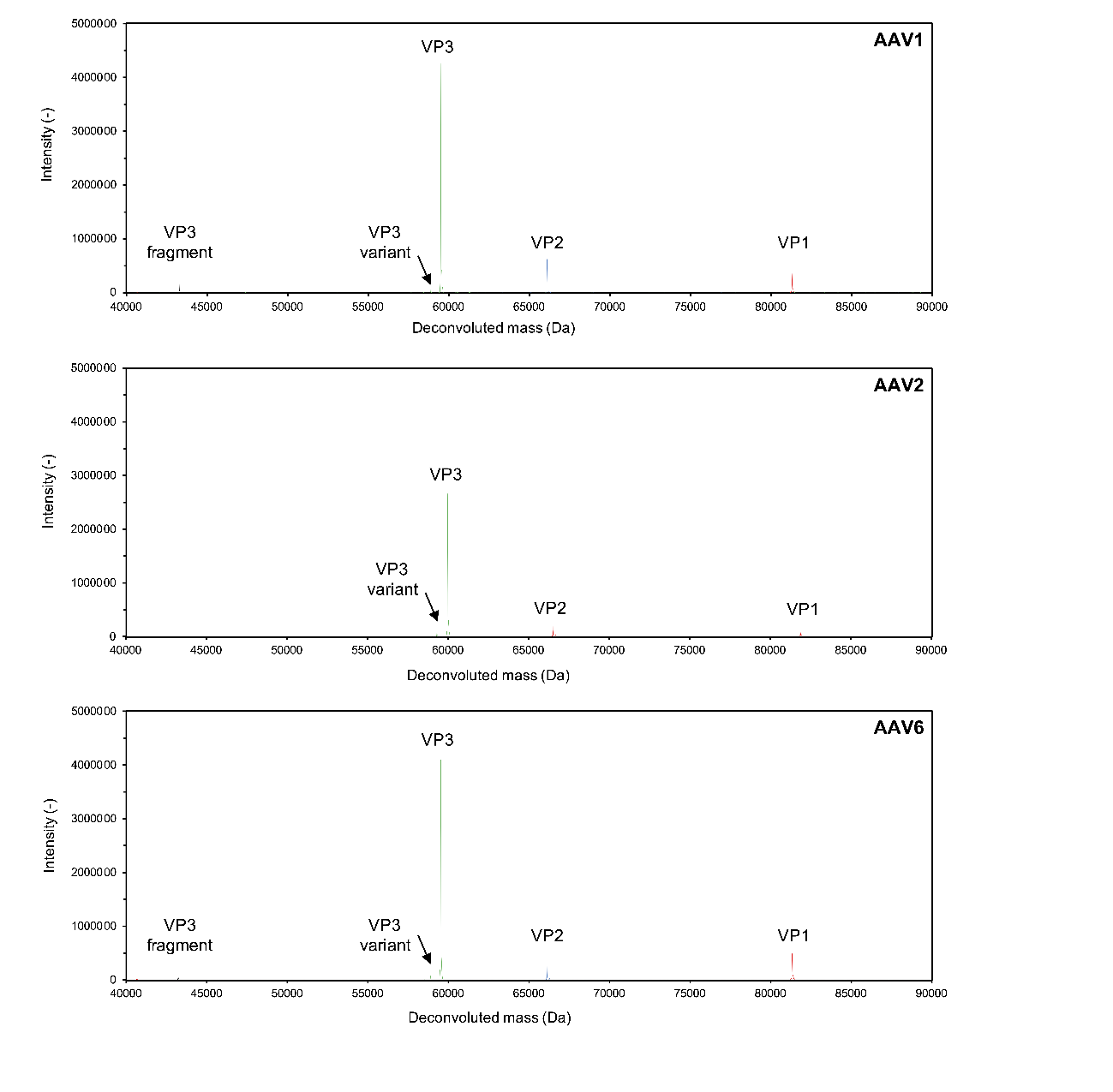

Supplement: Supplemental data [file Suppl_FigureS2.docx]
