# Supplementary material for: Characterization of Adeno-Associated Virus Capsid Proteins with Two Types of VP3-Related Components by Capillary Gel Electrophoresis and Mass Spectrometry
Source: Hum Gene Ther. 2021 Nov 15;32(21-22):1403–16. doi: 10.1089/hum.2021.009 (PMC10112878; doi:10.1089/hum.2021.009)

**Figure S4a.** DNA sequence alignment of VP3 of AAV1-12 and AAVrh10. AAV1, AAV2, AAV3, AAV6, AAV8, AAV10, and AAVrh10 have the second potential initiation codon besides the first common initiation codon for VP3. As described in the main text, the first initiation codon which has A in the -3 position besides G in the +4 position (A in the initiation codon AUG is counted as +1) is favorable in expression level. AAV4, AAV5, AAV9, AAV11, and AAV12 have only one initiation codon. AAV7 also has one initiation codon but the small population of VP3 variant of which the translation starts from GTG was reported by a previous study.^21^


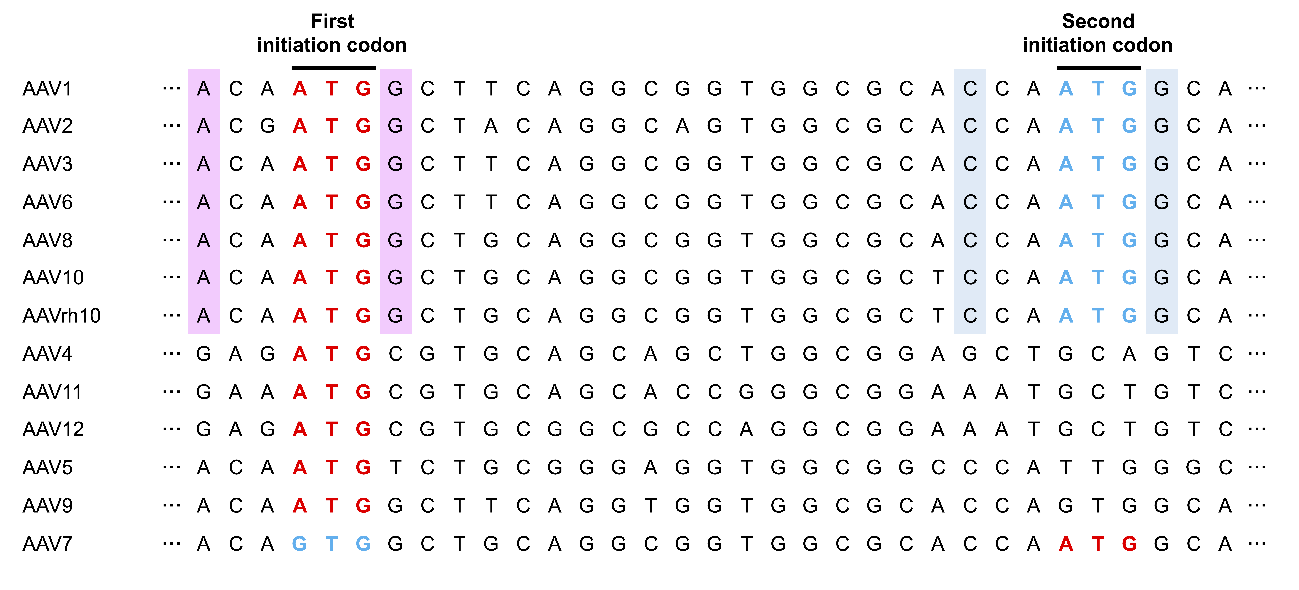


**Figure S4b.** Electropherogram of AAV5. AAV5 which has only one initiation codon shows no minor peak in the CGE result.


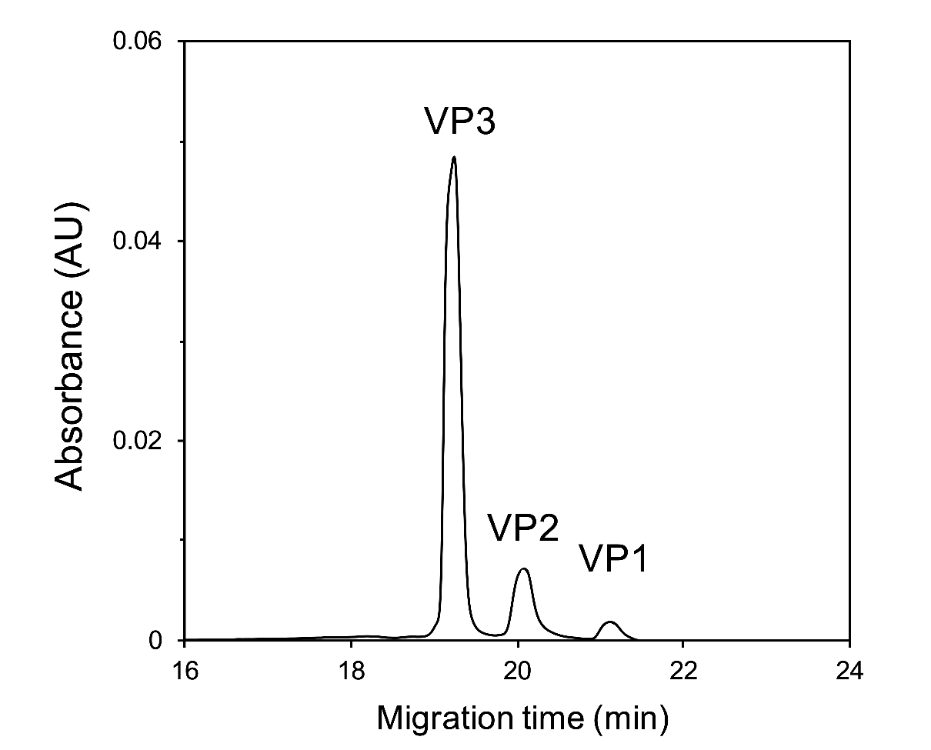


**Figure S5a.** LC-UV-MS result of AAV1 at room temperature (RT) at 25°C. The left figure is the LC chromatogram with the detection of intrinsic fluorescence. Black, red, and blue colors represent one replication: N1, N2, and N3, respectively. The right figure is the representative deconvoluted spectra of the VP3 peak using RT measurement of N1 data (black). The table shows the comparison of LC-UV-measurement between 80°C (Figure 4b, the column holding time is 0 min) and 25°C (This figure). In both of the LC-UV-MS measurements at 80°C and 25°C, the same amount of AAV1 is injected and the same LC conditions are employed. At RT, VP peaks can not be separated and only VP3 can be detected by deconvolution analysis. Considering that the peak intensity of VP3 is significantly decreased by 56.7-fold and the retention time is largely shifted as 8.3 min, it is necessary to apply a high temperature to obtain good separation of VP peaks.


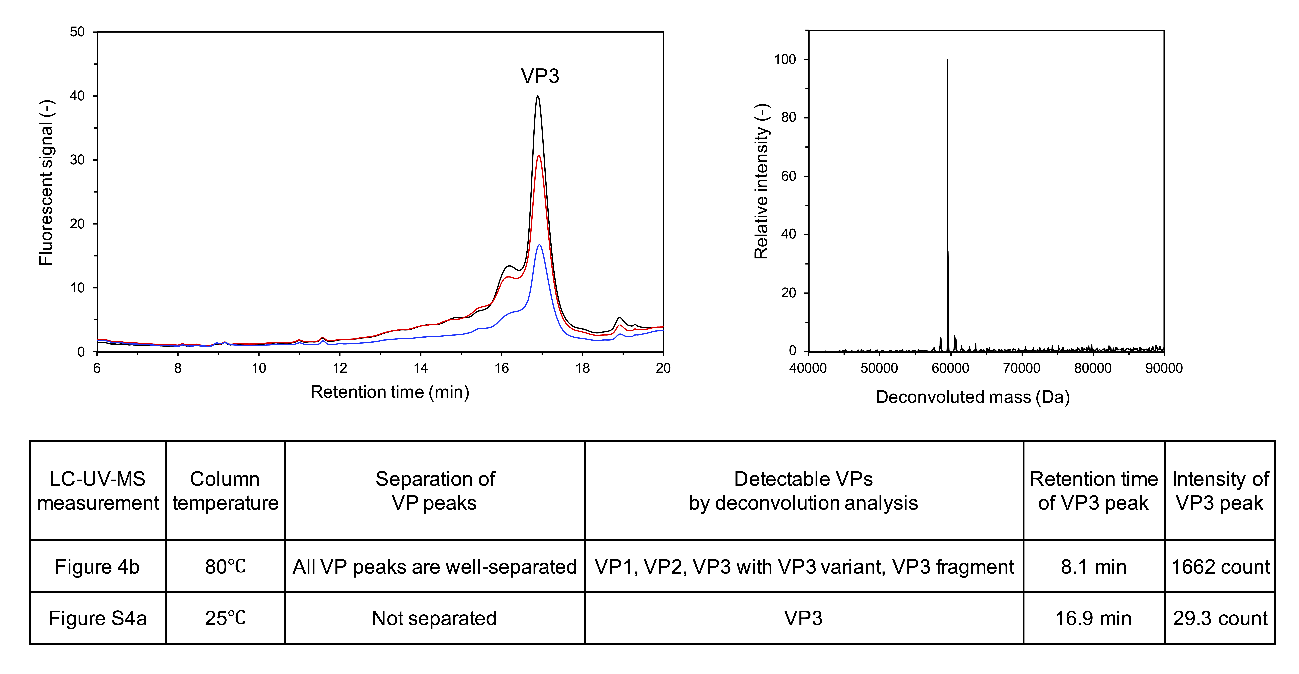

Supplement: Supplemental data [file Suppl_FigureS4.docx]
