# Supplementary material for: Characterization of Adeno-Associated Virus Capsid Proteins with Two Types of VP3-Related Components by Capillary Gel Electrophoresis and Mass Spectrometry
Source: Hum Gene Ther. 2021 Nov 15;32(21-22):1403–16. doi: 10.1089/hum.2021.009 (PMC10112878; doi:10.1089/hum.2021.009)

**Figure S5b.** LC-MS measurement of synthesized peptide, (a) total ion chromatogram (b) MS chromatogram of eluted peaks. Synthesized peptide has the same sequence as VP3 of AAV1 from F577 to D609 containing the specific DP site, “FGTVAVNFQSSSTDPATGDVHAMGALPGMVWQD”. Synthesized peptide is provided from Biologica Co. (Nagoya, Japan) with observed purity of >87%. LC-MS measurement was performed with a maXis II ETD ESI-QTOF mass spectrometer (Bruker) coupled with a Nexra HPLC (Shimadzu). The separation was performed on an ACQUITY BEH C18 column (300Å, 1.7 μm, 2.1 mm×150 mm, Waters) at a flow rate of 0.2 mL/min of DFA mobile phase and temperature of 80°C. The cleavage of DP sequence occurred with 6.1% of total peak area. Cleavage % of peptide is higher than VP3 possibly due to the high accessibility of DFA solution.


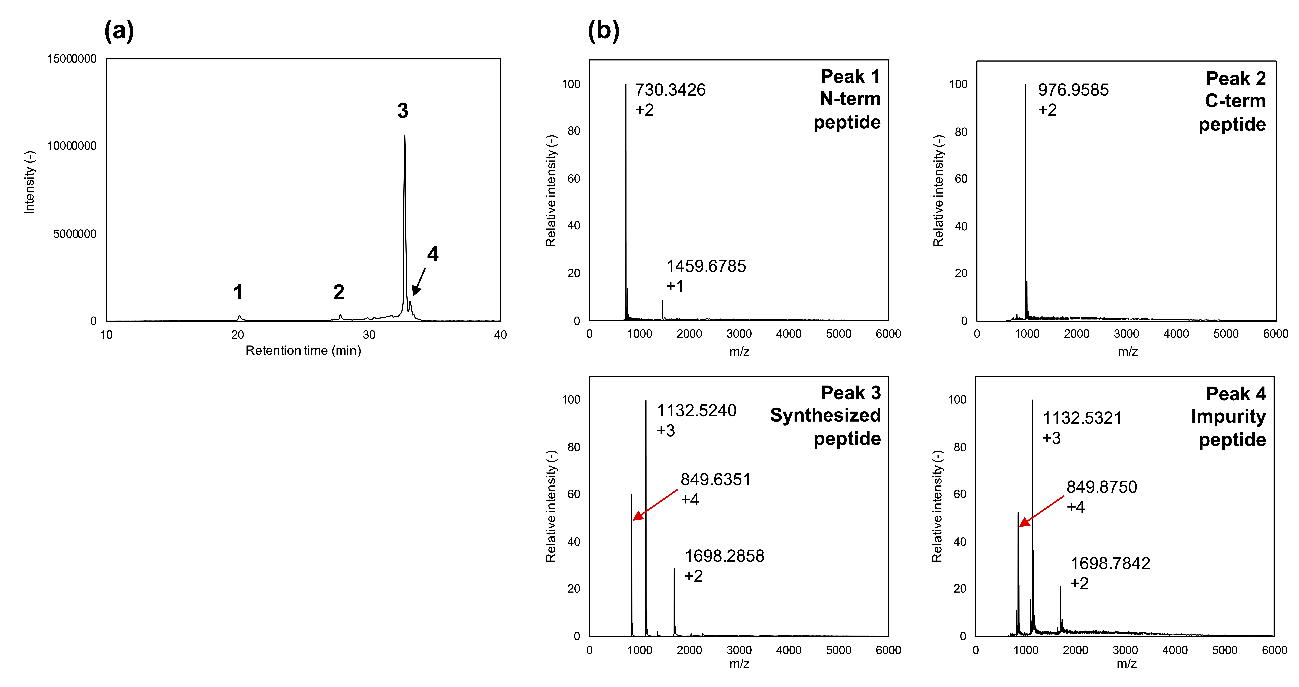


**Figure S5c.** Incubation test of AAV1 formulated in the elution buffer (pH 3.5) and 1x PBS (pH 7.4) until 72 h. Open circles and squares denote the elution buffer and PBS, respectively. As described in the main text, the LC-UV-MS method provides the imperative fragmentation of VP3, resulting in the generation of VP3 fragments which accounted for <3%. Since the peak area of VP3 fragment in all data point is <3% and there is no difference between the elution buffer and PBS, it is indicated that VP3 fragments are not produced in the elution buffer until 72 h.


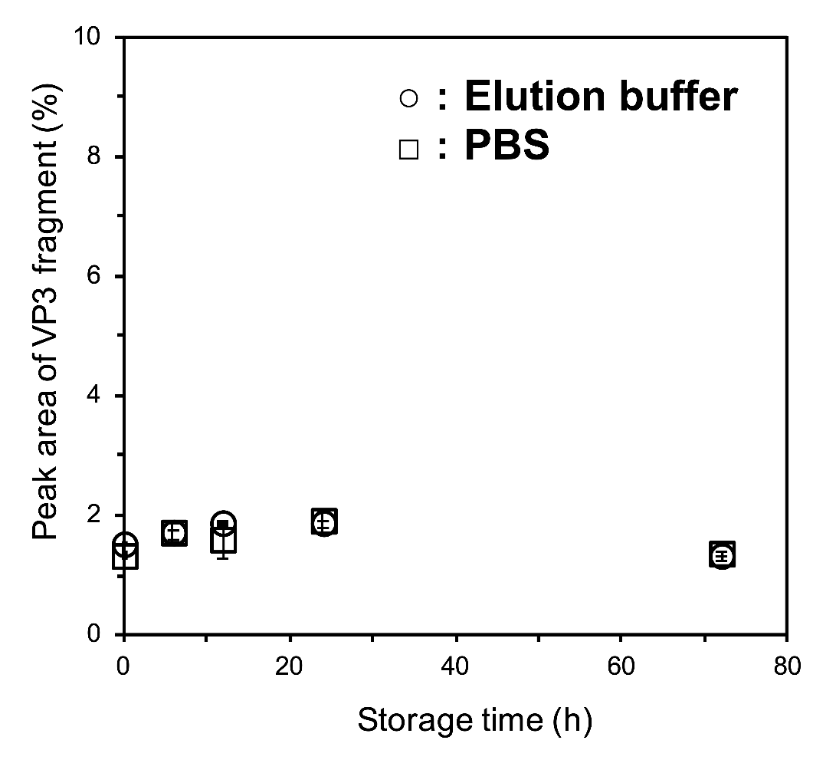

Supplement: Supplemental data [file Suppl_FigureS5.docx]
